# The effects of temperature on the biophysical properties of optic nerve F-fibres

**DOI:** 10.1038/s41598-020-69728-y

**Published:** 2020-07-29

**Authors:** Lavinia J. Austerschmidt, Azab Khan, Dafydd O. Plant, Ella M. B. Richards, Sophie Knott, Mark D. Baker

**Affiliations:** Centre for Neuroscience, Surgery and Trauma, Blizard Institute, QMUL, 4 Newark Street Whitechapel, London, E1 2AT UK

**Keywords:** Biophysics, Neuroscience, Physiology

## Abstract

In multiple sclerosis, exacerbation of symptoms with rising body temperature is associated with impulse conduction failure. The mechanism is not fully understood. Remarkably, normal optic nerve axons also show temperature dependent effects, with a fall in excitability with warming. Here we show two properties of optic nerve axons, accommodation and inward rectification (*I*_h_), respond to temperature changes in a manner consistent with a temperature dependent membrane potential. As we could find no evidence for the functional expression of K_V_7.2 in the axons, using the K^+^ channel blocker tetraethylammonium ions, we suggest this may explain the membrane potential lability. In order to understand how the axonal membrane potential may show temperature dependence, we have developed a hypothesis involving the electroneutral movement of Na^+^ ions across the axon membrane, that increases with increasing temperature with an appropriate *Q*_10_. Part, but probably not all, of the electroneutral Na^+^ movement is eliminated by removing extracellular Cl^−^ or exposure to bumetanide, consistent with the involvement of the transporter NKCC1. Numerical simulation suggests a change in membrane potential of − 15–20 mV mimics altering temperature between room and physiological in the largest axons.

## Introduction

Approximately 60 to 80% of patients with multiple sclerosis (MS, an autoimmune disease of the central nervous system causing demyelination), are affected by temperature sensitivity^1^. This includes the worsening of symptoms such as blurred vision, pain, motor problems or fatigue when the core temperature of patients rises. How these effects of temperature are mediated is not fully understood. The functional properties of central axons have remained less well defined than those in the periphery, in part because white-matter preparations are less amenable to experiments using threshold-tracking. However, knowledge of white-matter behaviour is required in order to answer fundamental questions about how some important symptoms of neurological disease are generated.

Recovery cycles measure the threshold changes occurring immediately after an action potential, and give an insight into the biophysical properties of optic nerve axons. In normal nerve they include a relative refractory period followed by a period of superexcitability (e.g.^2,3^). Threshold-tracking is a technique ideal for mapping out recovery cycles, where the properties of the refractory period depend in large part on the escape of Na^+^ channels from fast inactivation, and the superexcitable period depends on the internodal conductance, determined by voltage-gated channels including K^+^ channels (e.g.^4,5^), for these reasons both are very sensitive to changes in resting membrane potential.

Application of long polarizing currents to myelinated axons give rise to changes in membrane potential and therefore threshold, because they change the amplitude of the minimum stimulus necessary for exciting a response. The membrane potential changes resulting from the application of rectangular polarizing currents are called electrotonus, and the sequence of threshold changes that parallel the membrane potential changes are known as threshold-electrotonus (TE;^6,7^). Under circumstances where there are an abundance of operable Na^+^ channels, the changes in threshold parallel changes in membrane potential, and such TE data can be obtained from humans, non-invasively, and have been used to study the functional properties of axonal voltage-gated ion channels in patients, including those altered by mutation (e.g.^8^). By recording such threshold changes, studies in both human and rodent peripheral nerve have revealed otherwise inaccessible information about the state of the axons, and how this state can change, for example with changes in temperature or vascular perfusion.

Previously, we have reported altered biophysical parameters in optic nerve axons as the temperature of the preparation was changed, that are consistent with depolarization when the nerve undergoes cooling, and hyperpolarization when warmed^2,3^. Our present findings reveal that kinetically slow K^+^ channels (K_V_7.2) appear to have little role to play in generating accommodation in optic nerve F-fibres, very different from their role in the periphery^9^, and consistent with these channels being unimportant in setting resting potential. One aspect of the effects of warming already described, is that it is associated with a fall in membrane conductance, not an increase^2^, ruling out a major role for changes in K^+^ leakage in generating the phenomenon. We report that the amount of accommodation to a depolarizing current increases dramatically on cooling, supporting the presence of a temperature-dependent membrane potential in optic nerve axons^2,3^. Finally, we have shown that the pharmacological block of *I*_h_ appears to increase threshold when the axons are warm (33–35 °C), although not at room temperature (RT), suggesting that *I*_h_ helps set the resting potential of optic nerve F-fibres at physiological temperatures, limiting a latent temperature-dependent hyperpolarization.

## Results

### Recording recovery cycles in F-fibres

**Figure 1:**
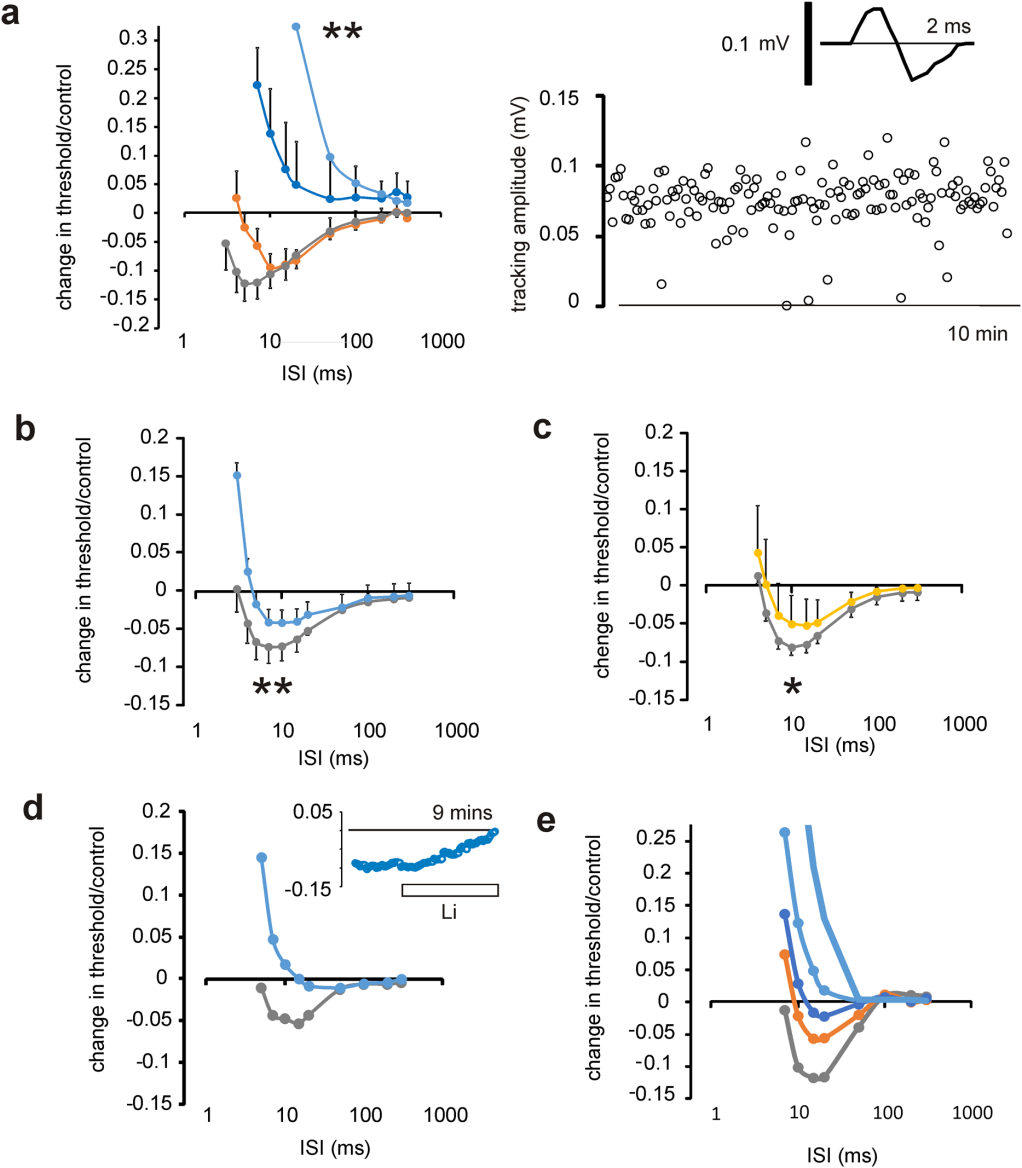
Recording recovery cycles in rat optic nerve shows increase in refractoriness with cooling, removal of Cl^−^ or reduction of [Na^+^]. (**a**), Single-unit recovery cycles at 22 (light blue), 26 (blue), 31 (orange) and 35 °C (grey), (*n* = 6,4,4,5, respectively) showing loss of superexcitability and an increase in refractoriness on cooling (left-hand panel). Error bars not shown on data for 22 °C for clarity, **p = 0.0018 (paired t-test, 22 vs. 35 °C). Right-hand panel shows continuous action potential responses over 10 min, with the amplitude occasionally dropping to zero when a sub-threshold stimulus current is applied. Inset, typical unit recording. (**b**), Compound F-fibre recovery-cycle responses in normal (grey) and Cl^−^ depleted solution (blue), showing decreased superexcitability and increased refractoriness with the loss of Cl^−^, **p = 0.002, paired-t-test (*n* = 5), 32 °C throughout. (**c**) Compared with normal (grey), 5 µM bumetanide increases refractoriness and decreases superexcitability (yellow) *p = 0.026, paired t-test (*n* = 4), 32 °C throughout. (**d**) Application of bumetanide followed by replacing extracellular Na^+^ with Li^+^ (light blue) produces a large increase in refractoriness similar to cooling to room temperature (control is grey, all at 33.5–34.5 °C; data representative of 8 experiments). Inset, real-time inhibition of superexcitability during perfusion of Li^+^ (**e**), Simulated recovery cycles, using the 2.1*P*Na simulation detailed in Table 1, and at 36 °C, show gradually increasing refractoriness at membrane potentials of − 97 (grey), − 92 (orange), − 90 (blue), and − 85 mV (light blue). Heavy light blue trace is at − 85 mV and 26 °C.

Recovery cycles are known to be a sensitive measure of axonal function, because the properties of the axons modifying the measurement of the cycle are critically dependent on membrane potential, including for example the kinetics of Na^+^ channel inactivation and the internodal conductance (e.g.^4,10^). Previously we have used compound F-fibre action potentials to record recovery cycles in isolated optic nerve, and while this has produced consistent information on the effects of temperature one possibility is that varying temporal dispersion of the units while changing temperature may obscure true changes in the cycle, through altered summation. We have addressed the issue by making recordings from whole optic nerve using the most minimal responses possible and have attained recordings of one unit (or a very few similar units recruited together) stably enough to generate recovery cycles, presented in Fig. 1a. One phenomenon that appears at this level are failures to excite any response during tracking, and coupled with the stereotypical response amplitude, this strongly suggests such recordings are of one or at most a few single-units. Under these experimental conditions the effect of temperature is not different from that previously found using compound action potential recording^2,3^, and shows large changes in refractory period that cannot be explained by the effect of temperature on gating kinetics alone, but rather is consistent with a temperature dependent membrane potential.

### Can removing extracellular Cl^−^ ions mimic the effect of blocking NKCC1?

In order to explain the temperature dependence of the membrane potential we have previously hypothesised that an electroneutral influx of Na^+^ exists that is associated with efflux carried by the electrogenic Na^+^ pump. We envisaged that the transporter NKCC1, that transports 1 Na^+^ and 1 K^+^ ion, accompanied by 2 Cl^−^ ions, was mediating part of the influx^2,3^. Further experiments in rat optic nerve (Fig. 1b,c), show that the removal of Cl^−^ and the effect of applying 5 µM bumetanide produce indistinguishable effects, reducing superexcitability and giving rise to a probable change in membrane potential of a few single millivolts, when compared with our simulated recovery cycles Fig. 1e. However, our experiments using cooling (such as in Fig. 1a) strongly suggest that there may be larger changes in membrane potential attainable than those produced by exposure to bumetanide, possibly implicating other electroneutral routes for Na^+^ movement that have not yet been identified. In order to discover whether this may be the case, and to uncover the full extent of the effects of Na^+^ transport on the axons, we have performed experiments with reduced Na^+^.

### Does reducing extracellular Na^+^ reveal likely effects on membrane potential?

If the hypothesis concerning electroneutral Na^+^ fluxes in optic nerve axons is correct, then reducing the extracellular Na^+^ ion concentration may reduce superexcitability and increase refractoriness, in a manner similar to cooling, by either reducing inward Na^+^ fluxes or by eliminating the standing return current generated by the Na^+^-pump (where the Na^+^-pump generates an outward current through the 3/2 exchange stoichiometry for internal Na^+^ and external K^+^ ions, respectively). Li^+^ is reported to be a substitute charge carrier for Na^+^ channels (e.g.^11^), but is much less well pumped out of cells with a *K*_0.5_ differing from that for Na^+^ by a factor of 4^12^. Reducing Na^+^ by equimolar replacement using Li^+^, may therefore inhibit both electroneutral Na^+^ movement and reduce the pump return current.

In our experiments using Li^+^ to replace Na^+^, we found a large loss of superexcitability, and the effects can be much larger than the effect of bumetanide alone (Fig. 1d). With such a modest reduction in Na^+^ the ability to track-threshold can be retained over long periods when bumetanide was used to block NKCC1 before Li^+^ application. Without pre-exposure to bumetanide, excitability was quite rapidly lost, within about 10 min—consistent with Li^+^ being transported on NKCC1, and building-up inside the axons (supplementary Fig. 1). The extracellular Na^+^ reduction we have used would be consistent with a drop in the transmembrane concentration gradient for Na^+^ of 30%, assuming intra-axonal Na^+^ is 10 mM. At the same time that would be expected to reduce *E*_Na_ by 14% (ie 10 mV), and the fall in the electrochemical gradient for Na^+^ near the threshold potential would be at most around 8%, and near the resting potential even less. Modest reductions in extracellular Na^+^ ion concentration would therefore be expected to have greater effects on electroneutral transport mechanisms than on current through Na^+^ channels. An inhibition of the pump-current would be expected if Li^+^ were transported into the axons instead of Na^+^ and our findings are consistent with the effect of exposure to ouabain already reported^2^.

### Simulation of recovery cycles

Recovery cycles (Fig. 1e) were generated using QTRAC-S (a threshold-tracking program, indicated in the methods, that has the facility to simulate axon behaviour) with the same parameter values used for simulations of TE (shown in Fig. 4) at 36 °C, detailed in Table 1. The cycles are plotted for a range of membrane potentials between − 97 and − 85 mV, and can mimic the disappearance of superexcitability and increase in refractoriness seen in our experiments.

**Table 1:** Changes in default parameters for Hugh Bostock’s model in QTRAC-S.

Parameter values at 36 °C and 26 °C	At 26 °C
*P*_Na_ 2.1 (cm^3 ^s^−1^ × 10^−9^)	A_αm_ = 0.81 (ms^−1^)
P_Nap_% = 0	A_βm_ = 0.037455 (ms^−1^)
*G*_Ks_N = 0.4 (nS)	A_αh_ = 0.004083 (ms^−1^)
*G*_Ks_I = 0.015 (nS)	A_βh_ = 0.279517 (ms^−1^)
*G*_Kfn_ = 500 (nS)	A_αn_ = 1.11666 (ms^−1^)
*G*_Kf_I = 1,000 (nS)	A_βn_ = 1.987 (ms^−1^)
*G*_lKn_ = 2.8 (nS)	A_αs_ = 0.41333 (ms^−1^)
*G*_lK_ = 3.2 (nS)	A_βs_ = 0.25037 (ms^−1^)
B_αh_ = − 110.1 (mV)	A_αq_, _βq_ = 0.24333 (ms^−1^)
B_βh_ = − 27.9 (mV)	B_q_ = − 133.1 (mV)
	At 36 °C
	A_αh_ = 0.01225 (ms^−1^)
	A_βh_ = 0.83855 (ms^−1^)

For simulation at 36 °C, default rate constant values were used, apart from those for *h*, where the rate constants were halved. For simulation at 26 °C, calculating from values used at 36 °C, Q_10_ for *m* is 2, Q_10_ for other A rate constants is 3. On simulating TE, resting membrane potential of nodes and internodes (*E*_NR_ and *E*_IR_, respectively) set to − 100 mV at 36 °C.

### Threshold-electrotonus (TE) in F-fibres

**Figure 2:**
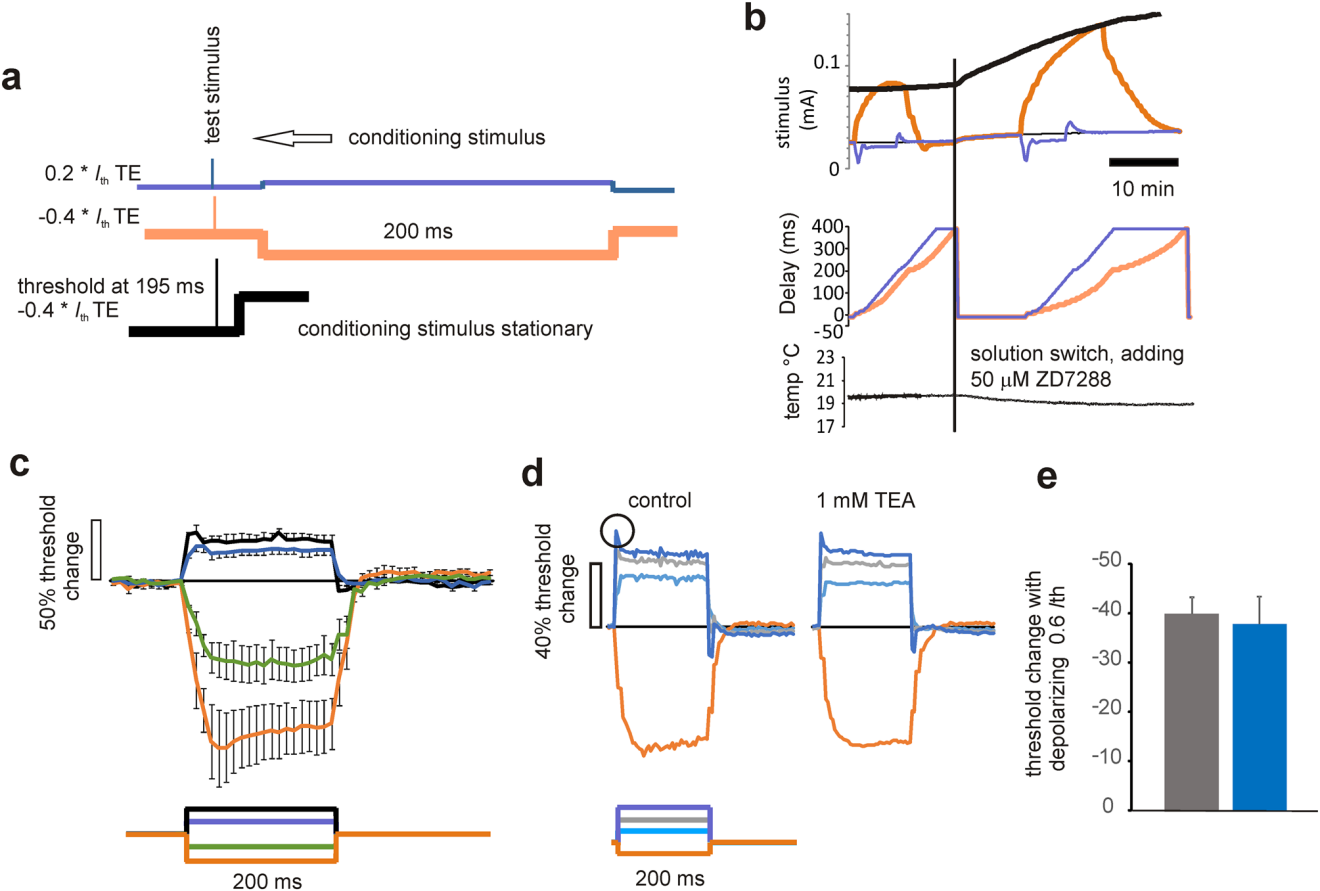
QTRAC current-threshold recording for TE experiments, with a bath solution switch and the addition of 50 µM ZD7288. (**a**), diagrammatic representation of three different protocols used to stimulate and polarize the F-fibres. Upper trace (blue) shows 0.2 * *I*_th_ conditioning stimulus (i.e. polarizing current), that is made to advance leftward across the 200 µs test stimulus when the tracking criteria have been fulfilled. Conditioning polarization 200 ms in duration. Middle trace (orange), − 0.4 * *I*_th_ hyperpolarizing conditioning polarization, similarly made to advance. Lower trace (black) shows threshold-tracking occurring at 195 ms into the − 0.4*I*_th_ polarizing current, and where the conditioning polarization does not advance. This latter situation gives a pseudo continuous plot of the final TE amplitude, that can be seen in (**b**). (**b**), Upper panel, raw threshold plot for control (fine black), 0.2 * *I*_th_ (blue), − 0.4 * *I*_th_ (orange). Thick black line, − 0.4 * *I*_th_, plot of threshold at 195 ms. Middle panel, plot of the time delay of the start of the polarizing current for the two protocols in (**a**). At time 0, the onset of polarization matches the time of the stimulus current. Lower plot temperature in °C in the bath next to the nerve. Threshold-electrotonus (TE) in F-fibres. (**c**) Responses to depolarizing and hyperpolarizing currents (inset below 0.2, 0.4, − 0.2, − 0.4 *I*_th_) suggests a greater internodal conductance in the depolarizing direction, consistent with outward rectification, with very little or no slow TE (*n* = 4). Slow TE is present in the hyperpolarizing direction, and at − 0.4 *I*_th_, there is the clear recruitment of inward rectification, *I*_h_. Temp 30–37 °C, *n* = 4. (**d**) Responses to depolarizing (0.2, 0.4, 0.6 *I*_th_) and hyperpolarizing (− 0.2 *I*_th_) currents shown. TEA insensitive accommodation is apparent when nerve is subject to largest depolarization (indicated by circle). (**e**) Threshold change at the end of depolarizing currents is unchanged by TEA (left and right columns, control and TEA, respectively; *n* = 3), indicating *G*K_S_, K_V_7, is not contributing to the accommodation. Temp = 32–35 °C.

TE was evoked by applying 200 ms duration sub-threshold polarizing currents at near physiological temperatures, and the depolarizing and hyperpolarizing currents gave rise to a fall in threshold and an increase in threshold, respectively, much as expected from numerous experiments on peripheral nerve (e.g.^6–8,13–15^), Fig. 2. The difference here is that these are the first such threshold recordings attempted from central axons. The asymmetry of the responses is consistent with outward rectification and the presence of kinetically fast voltage-gated K^+^ channels in the internodal membrane, and also the modification of hyperpolarizing responses by inward rectification, HCN *I*_h_ (e.g.^16^). The presence of both these conductances have been noted over many years in other experiments on optic nerve by different authors, including Gordon et al*.*^17^, Eng et al*.*^18^ and Devaux et al.^19^. We report there are significant differences from the responses in human peripheral nerve, notably the small slow TE in the depolarizing direction. A similar difference between human and rat peripheral nerve was recognized early, and is explained by the greater expression of fast K^+^ channels in the internode of rat nerve^6^.

### Effects of applying TEA ions

Key amongst the ion channels contributing to the control of peripheral nerve excitability is *G*_Ks_, K_V_7.2, KCNQ2^9,20–25^. These channels are kinetically slow, help set the membrane potential and the nodal membrane conductance, and limit repetitive firing (e.g.^9^). These channels are blocked by 1 mM tertaethylammonium (TEA) ions, outside the axons, and by Ba^+^ ions. TE in the periphery is modified by the activation of *G*_Ks_, which causes a prominent sag in the waveform as the activation of the channels limits the fall in threshold caused by depolarization (e.g.^7^). Devaux et al. 2004^23^ reported on the distribution of K_V_7.2, revealing it to be a nodal channel, but that its functionality in optic nerve disappeared with nerve maturation.

In order to discover whether *G*_Ks_ made a significant contribution to accommodation in F-fibres, we recorded TE in optic nerve at near physiological temperature (Fig. 2), and then exposed the axons to 1 mM TEA, allowing equilibration with the bath solution for 10 min. We can report that application of TEA did not change TE in any obvious way, and that measurements of the effect on accommodation were without substantial or significant effects (*p* = 0.453), Fig. 2c,d,e. However, we are aware that there is evidence for an effect of TEA on optic nerve fibres, but only after previous exposure to 4-aminopyridine (4-AP) that generated a widened action potential^17^. So these channels are likely to be there, but at least under the circumstances of our recordings, not functionally significant.

### Effects of ZD7288 application

**Figure 3:**
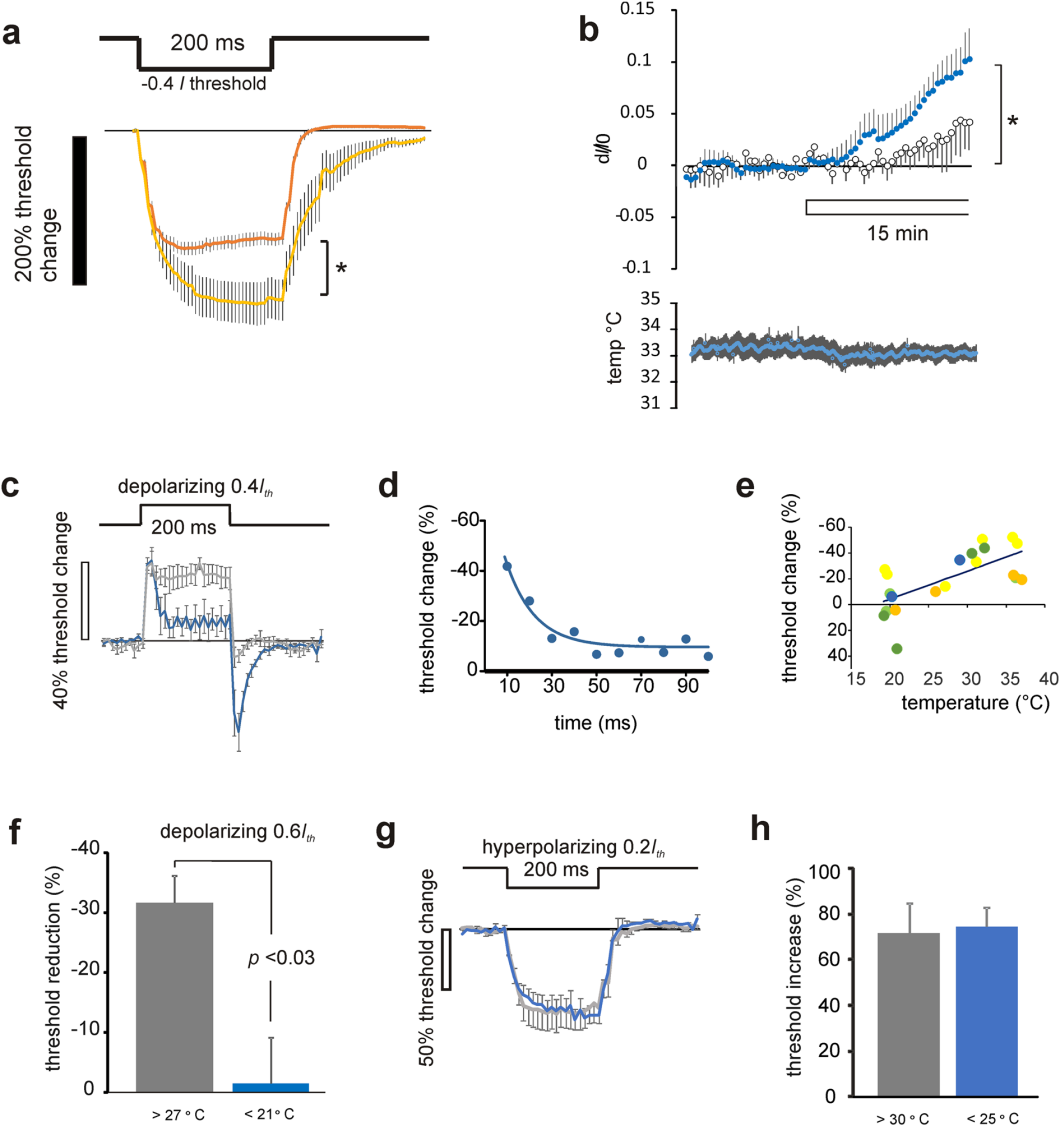
F-fibre TE is dramatically changed by altering temperature and exposure to ZD7288. (**a**) 50 µM ZD7288 alters hyperpolarizing TE and eliminates the rectification caused by *I*_h_ (orange and yellow traces, without and with ZD7288, respectively, **p = 0.028, paired-t-test, n = 6). (**b**) Application of 50 µM ZD7288 (shown by bar) increases the resting-threshold (dI/I0: change in threshold current over current at time 0, when drug first goes on, suggesting hyperpolarization (filled circles; *p = 0.013, single-sided t-test, n = 7; data normalized at time 0, when the drug first goes into the bath, and subtracting a sloping baseline; temp = 32–35 °C). Open circles, plot of change in control threshold at room temperature (n = 5), reveals a smaller increase in the average threshold, in comparison to that seen at warmer temperatures (ns). Lower panel, plot of bath temperature during the solution change in the warm, plotted as means ± SEM (n = 7). Depolarizing TE is dramatically changed by altering temperature**.** (**c**) Accommodation to a 0.4 *I*_th_ depolarization is enhanced by cooling (> 30 °C grey, compared with < 21 °C, blue, n = 4,5, respectively). (**d**) The mean threshold-change data on depolarization at room temperature are well described as an exponential with τ = 12.78 ms, consistent with the mechanism of accommodation being Na^+^ channel inactivation. (**e**) The threshold-change occurring at the end of a depolarizing step diminishes with cooling, slope of the plotted regression line (− 2.18) is significantly different from zero (**p = 0.0011, ANOVA). Data from each nerve colour coded. (**f**) The threshold reduction at the end of a depolarizing step is significantly reduced on cooling (n = 5; paired-t-test). (**g**) Responses to − 0.2 *I*_th_ polarizing current is similar at both cool (< 25 °C) and warm (> 30 °C) temperatures. (**h**) No change in the maximum average threshold increase (n = 5,4, respectively) recorded over 200 ms.

ZD7288 is pharmacological tool widely investigated as a blocker of anomalous rectification (HCN channels) in a variety of tissues. For example it is a blocker of *I*_h_ in nervous tissue (e.g.^26,27^) and *I*_f_ in the heart (e.g.^28^). We report that application of 50 µM ZD7288 when the optic nerve is warm (~ 35 °C) produces a block of the prominent sag on − 0.4 *I*_th_ TE waveform, and increases the recovery time from applied hyperpolarization (Fig. 3a), indicating a fall in the axonal membrane conductance, and is consistent with *I*_h_ operating at or near the resting potential. Also, an investigation of the effects of ZD7288 application on the resting threshold in the warm (Fig. 3b) reveals that the drug likely increased the threshold over a 15 min application (*p* = 0.013), consistent with *I*_h_ contributing to setting the axonal resting potential and limiting a latent temperature dependent hyperpolarization that we have already concluded must be present^2,3^. Evidence in other neuronal preparations has revealed the functional effects of *I*_h_ block on excitability are dependent on the value of the resting membrane potential. Where membrane potential is low in substantia nigra neurons (− 53 mV), block of *I*_h_ does not affect resting membrane properties^26^. In the present experiments we found that the effect on threshold at 15 min drug exposure was substantially reduced at room temperature, on average, from that found at 32–35 °C, although this reduction in change was not statistically significant (Fig. 3b)*.* At more negative membrane potentials and also with intrinsically varying membrane potentials the functional effects of *I*_h_ block are revealed more prominently^16,24,29^. The threshold increase we have seen at warm temperatures seems a reasonable match with the − 5 mV change in membrane potential reported by others and occurs consistently accompanied by minimal changes in temperature.

So far the data indicate that axonal threshold continues to rise in the presence of ZD7288, and hyperpolarizing TE continues to get bigger following drug exposure and this can go on for as long as 1 h, so the ultimate change in membrane potential brought about by exposure to ZD7288 might be larger than − 5 mV. We note that the temperature dependence of control threshold that we have already reported (that is U-shaped over the range of temperatures studied;^3^), can explain the small and non-incremental increase in threshold seen on the slight further cooling occurring with switching solutions in Fig. 2b. This type of effect is quite typical of optic nerve axons and helps demonstrate their acute temperature sensitivity.

### What are the effects of changing temperature on accommodation?

Our recordings indicate that there is no important TEA sensitive increase in threshold during sub-threshold depolarizations, although there appears to be a brief and very incomplete phase of accommodation occurring at the beginning of the largest (0.6 *I*_th_) depolarizing currents, that must be attributable to another mechanism. The simplest explanation for this is that it is owing to a partial inactivation of the Na^+^ channels at the greatest sub-threshold depolarization. Reductions in the numbers of operable Na^+^ channels increases threshold, and in certain circumstances, notably with chronic depolarization, the numbers of available Na^+^ channels can become critical (e.g. under conditions where anode-break excitation can occur;^7^). However, the clear finding is that there is very little accommodation at near body temperatures in the F-fibres. This situation dramatically changes when the nerve is cooled. In these circumstances the accommodative process gets larger with a greater disparity from physiological temperatures (Fig. 3c,e,f). This accommodative process has an exponential time-course, with a time-constant appropriate for the disappearance of Na^+^ channels into the fast inactivated state, as measured in patch-clamp (Na_V_1.6 at room temperature, and at a membrane potentials near between − 60 and − 80 mV (^30^, their Fig. 4;^31^) (Fig. 3d). Complete accommodation, such as that found for cool F-fibres, has previously been seen in the periphery, but only under conditions of ischaemia or high extracellular K^+^, and these findings can be explained if the cool F-fibres are depolarized.

**Figure 4:**
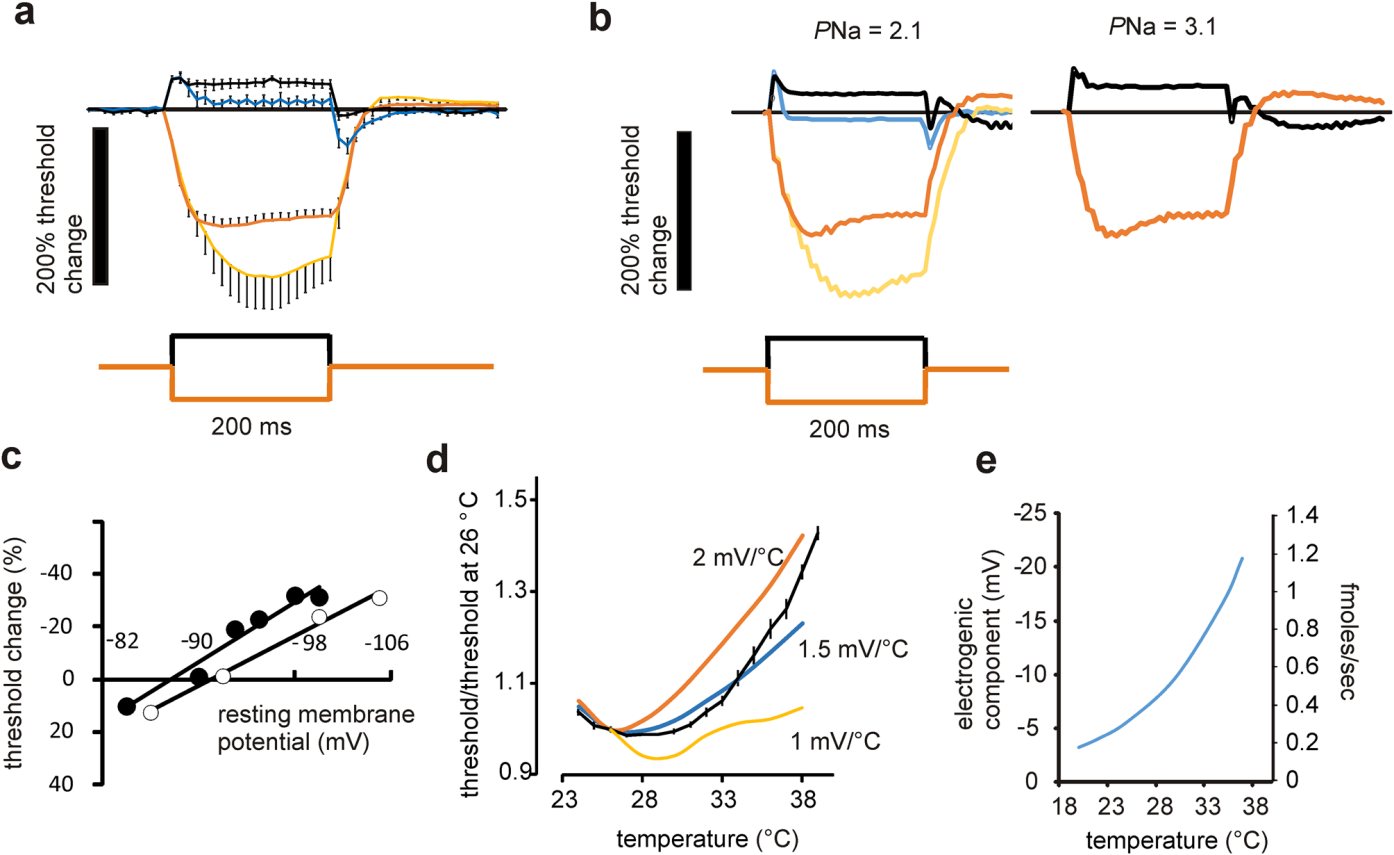
Changes in TE and threshold, with changing membrane potential and temperature, approximated by a simulation in QTRAC-S. (**a**) TE responses (average ± SEM) in F-fibres to ± 0.4 *I*_th_ at temperatures > 30 °C (black, orange), and < 25 °C (blue, yellow). Polarizing waveforms inset. (**b**) The simulated responses to ± 0.4 *I*_th_ polarization in QTRAC-S, where the resting membrane potential is − 100 mV and temperature 36 °C, − 88 mV and 26 °C, black and blue traces, respectively, showing increase in accommodation with cooling and allowing for a shift in the voltage-dependence of *I*_h_ activation at cool temperatures of − 30 mV. *P*Na variable set to 2.1. Responses are similar to the experimental records obtained in **a,** for > 30 °C. Right-hand panel, similar findings for 36 °C with *P*Na variable set to 3.1. (**c**) The number of Na^+^ channels incorporated impacts the potential at which the accommodation/membrane potential relation crosses the x-axis, indicating complete accommodation (filled circles, *P*Na = 3.1, open circles *P*Na = 2.1). (**d**) Changes in resting-threshold with changing temperature, with a membrane potential of − 85 mV at 26 °C, approximated by simulations in QTRAC-S. Real threshold measurements (average ± SEM) in F-fibres (black). Simulated threshold changes with 1, 1.5 and 2.0 mV/°C, plotted as continuous yellow, blue and orange curves. (**e**) The relation between the size of the electrogenic component of the membrane potential plotted against temperature, with a pump component of − 4 mV at 22 °C, and *Q*_10_ = 3 for electroneutral Na^+^ transport and exchange, for a length of axon 0.55 GΩ membrane resistance (see text).

Two possible mechanisms have been advanced to explain a temperature dependent membrane potential. One of the mechanisms relies on electroneutral Na^+^ influx^2^, while the other proposes a warming activated K^+^ conductance^32^. In order to test the possibility that TE might reveal evidence for a leakier nerve membrane with warming, we compared the threshold changes to 0.2 *I*_th_ hyperpolarizations (that activate *I*_h_ minimally), near room temperature and at near physiological temperatures and found, on average, no change in the responses of the axons (Fig. 3g,h).

### Mimicking TE responses using QTRAC-S simulation

We have used Hugh Bostock’s model axon in QTRAC-S to approach simulating the optic nerve F-fibres, and to approximate the effects of changing membrane potential on the TE responses. Our findings are summarised in Fig. 4, and the changes we made in the default variable values, to more adequately obtain responses similar to those of F-fibres, are shown in Table 1. First, steady-state depolarization can be seen to be responsible for the appearance of accommodation in real nerve (Fig. 4a) and in the simulation (Fig. 4b,c), and there is an almost linear relation between the extent of accommodation and the membrane potential (Fig. 4c), similar to the effect of temperature in the real axons (Fig. 3e). As the axons depolarize, the degree of accommodation increases. Further, we found that this effect was not critically dependent on assumptions related to initial conditions such as the absolute value of membrane potential at a particular temperature or the numbers of Na^+^ channels (Fig. 4c). On the contrary, the effect of depolarization on accommodation is robust. Second, in the depolarized condition it is possible to obtain markedly asymmetric TE waveforms (i.e. when comparing depolarizing and hyperpolarizing responses), where the hyperpolarizing waveform shows clear evidence for inward rectification (*I*_h_). This inward rectification appears attenuated in our recordings on cooling with slow TE becoming significantly larger (*p* = 0.045 Mann–Whitney U-test, *n* = 8,15, RT and 33–35 °C, respectively), an effect brought about in part by a slowed rate of *I*_h_ activation, and the deduced shift in resting membrane potential in the cool. In addition, the availability of *I*_h_ is likely reduced when the axons are in a depolarized state (possibly through the effect of modal gating in HCN1 channels, discussed below;^33^).

## Discussion

The temperature dependent completeness of accommodation provides new evidence that F-fibre axons have temperature dependent membrane potentials; the membrane potential must decrease on cooling, and increase on warming. The observation that accommodation becomes significantly more complete when the nerve is cool mimics the effect of depolarization first studied in peripheral axons by Baker and Bostock^7^. The idea that optic nerve axons have temperature dependent membrane potentials was proposed by Coates et al*.*^2^, and the mechanism suggested was one requiring electroneutral Na^+^ entry in to axons, that increases with rising temperature and is countered by electrogenic expulsion by the Na^+^-pump. An earlier idea (e.g.^32^), that temperature dependence is brought about by an increase in K^+^ conductance on warming we previously rejected, because of the effect of temperature on the depolarizing afterpotential in optic nerve^2^. While TE is critically dependent on the availability of Na^+^ channels, and is therefore affected by processes other than those modifying membrane conductance, the present experiments do not help make a case for an increase in the resting membrane conductance with warming. Negative polarizing currents at − 0.2 *I*_th_ (that minimally activate *I*_h_) do not evoke less change in threshold with warming, that would be expected with a leakier membrane, in fact over 200 ms there is no difference in the effect of polarization with warming. These findings are consistent with our original conclusion that the temperature dependent effect is brought about without an increase in resting conductance.

Simulation (Fig. 4c) suggests a change in membrane potential of the order of 15–20 mV for these axons mimics altering temperature between room and physiological, with the cool axons being more depolarized. This value is smaller than the change in potential estimated from demarcation potential recordings off whole optic nerve (− 40 mV from room to physiological^2^), and changes in potential observed in patch-clamped pyramidal neurons^32^, that provide similar values. That said, the F-fibres represent the axons with the highest conduction velocity and are likely the largest in the optic nerve. We therefore predict that the change in membrane potential in the smaller axons for the same temperature swing may be larger.

Kanagaratnam et al.^3^ reported that the relationship between the resting threshold and axonal temperature is a ‘U’ shape, and we have generated a simulation showing the ‘U’ using the same assumptions as that shown in Fig. 4, (with P_Na_ = 2.1). The insights gained from this simulation are shown in Fig. 4d. Notably, when the temperature increases from close to 26 °C the resting membrane potential is changed by between 1.5 and 2 mV/°C, producing increases in threshold that are very similar to those found. Finally, how might the increase of threshold from 26 °C with cooling be explained? Here we suggest there are likely two explanations. Firstly, cooling the axons means that the Na^+^ channel activation kinetics slow down, and the channels cannot respond to a brief current pulse. This would mean that the amount of stimulus current would have to increase to produce the same axonal recruitment. Secondly, depolarization caused by cooling will increase the amount resting Na^+^ channel inactivation through its action on membrane potential. Sequestering Na^+^ channels into the inactivated state will cause threshold to rise because fewer channels remain operable.

In order to calculate resting energy expenditure caused by Na^+^ leaking into axons, Attwell and colleagues have made the assumption that the only source of Na^+^ is the leak through ion channels, and Equation 4 of Attwell and Laughlin^34^, also subsequently Equation 1 of Harris and Attwell^35^, allowed the calculation of ATP utilization to reverse this leak for a 5.5 mm length of optic nerve fibre. However, ATP expenditure on signalling in the neocortex, including that used for maintaining neuronal resting potential, appear to be calculated as a value somewhat less than experimental measurements^35^.

Where Na^+^ leakage into axons comes electroneutrally, and assuming that at steady-state, the intracellular Na^+^ ion concentration is unchanging and not dependent on the temperature (necessitating equal and opposite transport/exchange and pumping of Na^+^), and the required movement of 3 Na^+^ ions per single charge moved across the axolemma by the pump applies, the following relation exists at a given temperature t:

1 $$V = - \left( {{\text{X}}_{{\text{t}}} Fr} \right)/{3}$$

where *V* is the electrogenic component of the membrane potential dependent on electroneutral influx in volts, *X*_t_ is the number of moles/sec of Na^+^ entering per unit length of axon at a given temperature, *F* is Faraday’s constant, *r* is the membrane resistance of the unit length of axon in ohms. In order to account for a resting electrogenic component of − 20 mV (expected at physiological temperatures for the largest optic nerve axons), generated across a length of axon of transmembrane resistance 0.55 GΩ^35^, would require a flux of Na^+^ of at least 1.13 fmol/s, 4 to 5 times larger than that predicted by Harris and Attwell’s Eq. (1), and therefore utilizing more ATP to reverse.

The relation between the size of the electrogenic component (dependent only on electroneutral influx of Na^+^) with respect to temperature, assuming that the pump component of the resting potential at RT (22 °C) is − 4 mV, and allowing a *Q*_10_ of 3 for Na^+^ transport, is shown in Fig. 4e and based on Eq. (1). The functional significance of an electroneutral flux of Na^+^ into axons, summarized in Eq. (1), implies that warming will produce an electrogenic hyperpolarization that is limited by the membrane conductance. Certainly, as the membrane conductance increases with the hyperpolarization dependent increase in *I*_h_ activation, this will tend to limit the achieved steady-state hyperpolarization. One further prediction is that the activation of *I*_h_ will tend to linearize the change in membrane potential with increasing temperature. This approach may help to explain why almost all energy expenditure in central white-matter is said to be associated with the resting potential, not action potential signalling. Finally, blocking electroneutral Na^+^ entry may be neuroprotective because it will tend to increase energy reserves.

In peripheral axons, the TEA sensitive *G*_Ks_, K_V_7.2 channels are known to play a major role in setting the resting potential of peripheral axons, and this is because exposure to TEA gives rise to a depolarization^9^. Furthermore, TEA sensitive K^+^ channels have a profound effect on depolarizing electrotonus and in normally polarized axons form the major mechanism of accommodation^7,9^. In F-fibres, this now appears not to be the case, because there is no functional accommodation blocked by TEA, as shown here, and there is also no late subexcitability in F-fibre recovery cycles, shown by Kanagaratnam et al*.*^3^. These characteristics would be consistent with an axonal resting membrane potential that is less stable than that of peripheral nerve. At present we have not explored the degree to which 4-aminopyridine sensitive K^+^ channels in optic nerve axons are responsible for setting the resting potential, but it seems likely that such K_V_1 channels will behave in much the same way they do in the periphery and are therefore unlikely to contribute much, unless the axon is depolarized. Instead, they will normally prevent a slow depolarizing TE (consistent with the real and simulated depolarizing waveforms having no slow phase in the depolarizing direction) and modulate the amplitude of the depolarizing after potential. One possibility is that the resting potential of the central axon is set by a pump-current that increases with warming, as previously envisaged, with *I*_h_ providing the likely limitation, as we supposed for peripheral nerve following tetanic activation^9^, confirming a function suggested by Mayer and Westbrook^16^. Our observations with Cl^−^ and Na^+^ replacement reveal that the optic nerve axon membrane potential must be dependent on the extracellular concentrations of these ions, ie the membrane potential is both Na^+^ and Cl^−^ dependent, but our hypothesis would suggest primarily an indirect mechanism based on electroneutral co-transport (although the data on Cl^−^ removal might be consistent with a resting Cl^−^ permeability that on Cl^−^ removal can coincidently mimic the effect of bumetanide).

We conclude that the predominant method of accommodation in F-fibres, over the range of temperatures we have used in our experiments, is Na^+^ channel inactivation. This means that the number of operable Na^+^ channels during and at the end of a depolarizing pulse becomes a critical determinant of threshold, and we conclude this for several reasons. Firstly, the observed rate of accommodation is appropriate for measurements of inactivation time-constant made on heterologously expressed Na_V_1.6 in voltage-clamp^30^. Secondly, an insight from the simulation is that the potential at which accommodation becomes complete is dependent on the number of Na^+^ channels incorporated into the model. More Na^+^ channels means that complete accommodation occurs at a more depolarized potential, consistent with a requirement for a greater steady-state inactivation. Finally, this proposed mechanism of accommodation in the cool is the same as that proposed for peripheral nerve in a depolarized state^7^.

As we attempted to understand the effect of cooling on *I*_h_, it became clear that we could not produce a TE waveform like that recorded, by slowing the rate of *I*_h_ activation only, using a plausible gating *Q*_10_. Two simple strategies could be used to get a more convincing approximation, constrained by the properties of the existing QTRAC-S simulation. One was to assume that those axons contributing to the TE in the hyperpolarizing direction had lower membrane potentials (more depolarized at rest) than those contributing to the depolarizing TE. The second possibility was that *I*_h_ becomes less available in the cool nerve. The first idea works to reduce the appearance of *I*_h_ because the axons are at a resting potential further from the activation threshold for *I*_h_, but requires one to believe that different subsets of F-fibres would be recruited for depolarizing and hyperpolarizing TE. The second option works because there is less *I*_h_ operating, and attempts to use this method are shown in more detail in Fig. 4. We favour this latter approach at present not least because of its simplicity, but also it is a way of representing a voltage-hysteresis effect for channel gating described for heterologously expressed HCN1^33^, where a depolarized holding potential can flip the channel gating-mode into one that requires much greater hyperpolarization to activate the channel, and we hypothesize that such a process could be operating in optic nerve in the cool and may explain our findings. One final possibility is that there are simply fewer operating HCN channels in the cool.

Most people with multiple sclerosis (MS) have temperature dependent symptoms, meaning that existing symptoms, such as muscle weakness, get worse or new symptoms appear when core body temperature rises. This characteristic has been noticed over 100 years, and the now classical explanation is that where impulse conduction safety factor is low in the central nervous system, raising core body temperature causes impulse conduction to fail by making action currents too brief (e.g.^36,37^). A shorter duration action current must result from changed gating kinetics of Na^+^ channels^38^, although the electrical capacity of the damaged axon is not altered by the temperature changes and any K^+^ channels exposed in a denuded axon will respond more quickly to depolarization. This understanding has led to the exploration of therapeutic avenues including widening action potentials, by blocking fast K^+^ channels using 4-AP, in order to improve safety factor (e.g.^36^). One caveat to this logic is that whatever the combination of mechanisms causing the temperature dependence in the central nervous system, the result is a situation where changes in temperature limited to a fraction of a °C only can have substantial effects. Now we put forward another factor that may be key in generating temperature sensitive symptoms, and that is the normal temperature dependence of central axons. This is the tendency to have reduced excitability in axons, caused by hyperpolarization, when the temperature increases^3^. Blurring of vision in MS is present with optic neuritis, or inflammation of the optic nerve, and resulting demyelination. In the worst case, damage to axons can give rise to patches of blindness, and are associated with reduced visual evoked potentials revealing a loss of impulse conduction in the visual pathway. Where damage to axons is not enough to completely eliminate impulse conduction, but where it reduces the probability that impulses will propagate normally, any process that increases threshold, including a hyperpolarization, will tend to make impulse conduction slow down or fail. As we have associated warming with a membrane potential hyperpolarization, we suggest that a core body temperature rise may block impulse conduction by reducing the excitability of the optic nerve axons through this mechanism. One possibility is that the temperature dependence we have described may underlie at least part of the difference in temperature dependence between the peripheral and central nervous systems, and contribute to the conduction failure with demyelination in MS.

## Methods

### Animals

The study was carried out in the laboratories of QMUL, Barts and the London Medical School, where the animals were housed in a Home Office designated establishment under veterinary supervised conditions, with food and water ad-libitum. Adult male Wistar rats (~ 320 g), obtained from an approved supplier, Charles River Laboratories (Manston, Kent), underwent euthanasia by exposure to a rising concentration of CO_2_ followed by cervical dislocation, in accordance with the guidelines and regulations, and this includes ethical permission, detailed in the Schedule 1 protocol list approved by the Home Office UK legislation (Scientific Procedures Act 1986).

### Tissue preparation and electrophysiology

Optic nerves were quickly isolated, placed in oxygenated buffer and cleaned of adherent tissue, and mounted in a two-chambered nerve bath for ex vivo study^2^. The right-hand chamber in which almost all the optic nerve was positioned, was continuously perfused with oxygenated buffer solution. We could introduce drugs into this solution, and control the perfusion temperature using a heating wand (HPT-2 ALA scientific, Farmingdale, NY, USA), with continuous monitoring of the temperature by a thermistor placed near the nerve (TC10 npi electronic, Tamm, Germany).

Optic nerve represents an isolatable tract of white matter that can be studied ex vivo. We have used the threshold-tracking technique to investigate further an apparent difference between central and peripheral axons, as central axons have been proposed to be far more sensitive to changes in temperature^2,3^. A computer controlled, constant-current stimulator (Digitimer DS4, Welwyn Garden City, UK) was used to polarize and stimulate the nerve through a PVC suction electrode. Control of polarizing and stimulus currents was achieved using a PC running QTRAC-S (Hugh Bostock, Institute of Neurology; available through Digitimer). The stimulus duration was 200 µs, in order to allow adequate discrimination of the stimulus artefact and response. The response amplitude was maintained at close to 5% of the supramaximal response, and this corresponded to an action potential attributable to the F-fibres in the nerve.

### Recovery cycles

For recovery cycles using compound responses the test stimulus was adjusted by the computer to give a 5% supramaximal response amplitude^3^, and during a recovery cycle recording, the delay between conditioning and test stimuli was incrementally increased, once the response error was within 10% of the target response amplitude. For recording ‘near single-unit’ responses, the amplitude of the target response was reduced until the tracking program picked-up zero amplitude responses following stimulation—indicating that the modulated stimulus sometimes failed to excite any units whatever. The tendency to generate null responses we accepted as evidence that the tracking program was activating only one or at most a few similar units when a response was found.

### Threshold electrotonus

The test stimulus was adjusted by the computer to give a 5% supramaximal response amplitude, and then polarising currents were applied 200 ms in duration and up to ± 0.6 threshold-current (*I*_th_) amplitude.

Inbuilt filtering in the Warner instruments amplifier gave a band-pass between 0.1 and 1 kHz, and the recorded action potentials were sampled at 10 kHz. Careful examination of our recordings revealed no evidence for the polarising currents themselves evoking firing activity in the axons undergoing threshold-tracking.

### Buffer solutions used were as follows

Control solution contained (in mM): NaCl 140, HEPES hemi Na 10, CaCl_2_ 2.1, MgCl_2_ 2.12, KCl 2.5, Glucose 10. The Cl^−^ depleted solution contained: Na-Isethionate 140, HEPES hemi Na 10, CaCl_2_ 2.1, MgCl_2_ 2.12, KCl 2.5, Glucose 10. The reduced Na^+^ solution contained: NaCl 101.4, LiCl 38.6, HEPES hemi Na 10, CaCl_2_ 2.1, MgCl_2_ 2.12, KCl 2.5, Glucose 10. All solutions were buffered to pH 7.2–7.3 with the addition of small quantities of HCl if necessary.

Tetraethylammonium (TEA) chloride (Sigma,-Aldrich, Poole, Dorset, UK) was added to the perfusion buffer and made up from powder each experimental day. 4-Ethylphenylamino-1,2-dimethyl-6-methylaminopyrimidinium chloride (ZD7288) and bumetanide were obtained from Tocris (Bio-techne Ltd, Abingdon, Oxfordshire, UK), and made up as a stock solution of 50 mM and 5 mM, respectively, in DMSO, and stored at − 20 °C, allowing subsequent serial dilution into perfusion buffer on each experimental day. Final concentration of vehicle never exceeded 0.1%. We did test vehicle alone, and observed no associated changes in threshold.

### Data analysis

After identifying periods of polarization during the conditioned stimulus tracking record, the threshold electrotonus (TE) was generated by calculating the differences between the conditioned and control threshold recorded on alternate sweeps, and dividing through by the value of the control threshold obtained immediately before the conditioned threshold measurement. While this ratiometric method used for TE is intrinsically stable, finding changes in the control threshold alone, for example when altered by application of ZD7288 sometimes required linear baseline correction, based on the values of pre-drug exposure threshold.

### Statistical analysis

ANOVA, paired-t test, single-sided t-test or Mann–Whitney U-test were undertaken in order to determine statistical significance, with the critical value of *p* taken as 0.05. Where possible experimental values are quoted or plotted as means ± SEM.

## Supplementary information

Supplementary figure.

## Data Availability

The datasets generated during and/or analysed during the current study are available from the corresponding author at reasonable request.

## Abbreviations

ZD7288: 4-Ethylphenylamino-1,2-dimethyl-6-methylaminopyrimidinium chloride
TEA Cl: Tetraethylammonium chloride
4-AP: 4-Aminopyridine
LiCl: Lithium chloride
