# Supplementary material for: The effects of temperature on the biophysical properties of optic nerve F-fibres
Source: Sci Rep. 2020 Jul 29;10:12755. doi: 10.1038/s41598-020-69728-y (PMC7391707; doi:10.1038/s41598-020-69728-y)
Supplement: Supplementary file 1 — Supplementary figure. [file 41598_2020_69728_MOESM1_ESM.pdf]

The effects of temperature on the biophysical properties of optic nerve F-fibres

Lavinia J Austerschmidt, Azab Khan, Dafydd O Plant, Ella MB Richards, Sophie Knott and Mark D Baker

Supplementary figure 1

a

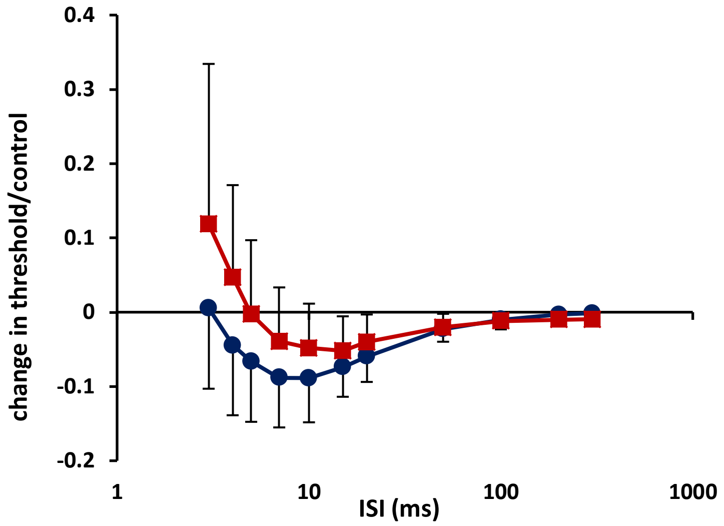

b

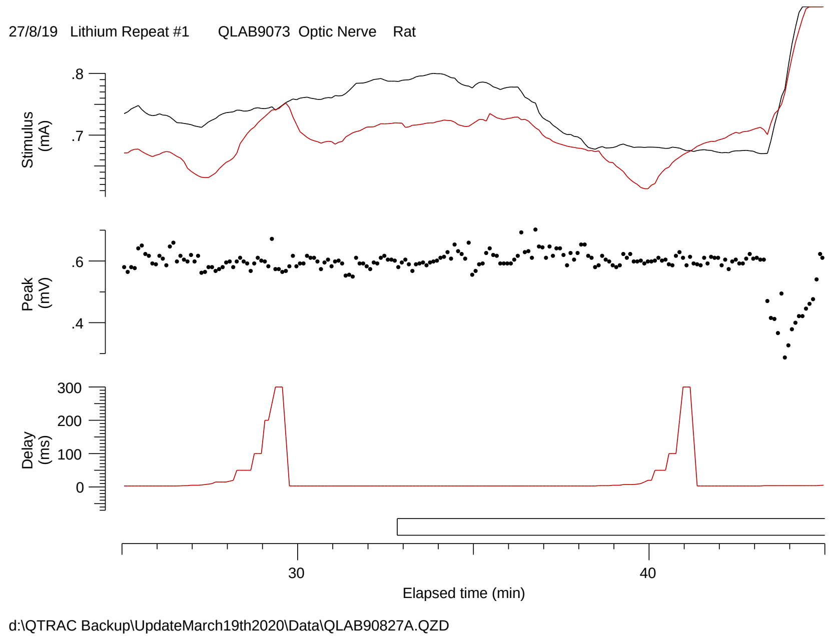

Supplementary figure 1. Effects of partial replacement of extracellular Na^+ with Li^+. a. Effects of Li^+ superfusion on the recovery cycle, recorded during the first ten minutes of solution exchange. Li^+ produces a significant increase in refractoriness and reduction in superexcitability, consistent with axonal depolarization, (control blue circles, in Li^+ red squares), means ± sem p=0.009, n=4, paired t-test, bath temperature 32 °C. b. Plot from QTRAC for Li^+ perfusion (indicated by open bar). Li^+ causes the loss of excitability during superfusion with falling action potential amplitude (middle panel) at around 43 minutes, and steep increase in thresholds at the same time (upper panel) black control, red conditioned. The variable delay between conditioning and test stimuli is shown in the lower panel in ms. Note, pre-exposure with bumetanide, prevents the loss of the action potential (see Fig 1 and text).
